# Development and evaluation of a multi-epitope subunit vaccine against group B *Streptococcus* infection

**DOI:** 10.1080/22221751.2022.2122585

**Published:** 2022-09-29

**Authors:** Yumin Zhang, Song Liang, Shiyu Zhang, Shidan Zhang, Yong Yu, Huochun Yao, Yongjie Liu, Wei Zhang, Guangjin Liu

**Affiliations:** a College of Veterinary Medicine, Nanjing Agricultural University, Nanjing, People’s Republic of China; b OIE Reference Lab for Swine Streptococcosis, College of Veterinary Medicine, Nanjing Agricultural University, Nanjing, People's Republic of China; c Joint International Research Laboratory of Animal Health and Food Safety, College of Veterinary Medicine, Nanjing Agricultural University, Nanjing, People's Republic of China; d Key Laboratory of Animal Bacteriology, Ministry of Agriculture, College of Veterinary Medicine, Nanjing Agricultural University, Nanjing, People's Republic of China; e Sanya Institute of Nanjing Agricultural University, Nanjing Agricultural University, Sanya, People’s Republic of China

**Keywords:** Group B *Streptococcus*, immunoinformatic, multi-epitope vaccine, immune protection, B cell epitope, T cell epitope

## Abstract

*Streptococcus agalactiae* (Group B *Streptococcus*, GBS) is a multi-host pathogen, even causing life-threatening infections in newborns. Vaccination with GBS crossed serotypes vaccine is one of the best options for long-term infection control. Here we built a comprehensive *in silico* epitope-prediction workflow pipeline to design a multivalent multiepitope-based subunit vaccine containing 11 epitopes against *Streptococcus agalactiae* (MVSA). All epitopes in MVSA came from the proteins which were antigenic-confirmed, virulent-associated, surface-exposed and conserved in ten GBS serotypes. The *in-silico* analysis showed MVSA had potential to evoke strong immune responses and enable worldwide population coverage. To validate MVSA protection efficacy against GBS infection, immune protection experiments were performed in a mouse model. Importantly, MVSA induced a high titre of antibodies, significant proliferation of mice splenocytes and elicited strong protection against lethal-dose challenge with a survival rate of 100% in mice after three vaccinations. Meanwhile, the polyclonal antibody against MVSA did not only inhibit for growth of GBS from six crucial serotypes *in vitro,* but also protect 100% naive mice from GBS lethal challenge. These active and passive immunity assay results suggested that MVSA could therefore be an efficacious multi-epitope vaccine against GBS infection.

## Introduction

*Streptococcus agalactiae*, also named group B *Streptococcus* (GBS), is a Gram-positive bacterium that can infect a wide range of species, including mammals, fish, reptiles, amphibians and birds [1]. This pathogen was currently divided into 10 serotypes (Ia, Ib, II–IX) based on the capsular polysaccharide, while six crucial serotypes (Ia, Ib, II, III, V, VI) were the most widely distributed [2]. GBS has been associated with over 500,000 preterm births each year, resulting in approximately 100,000 newborn deaths, at least 46,000 stillbirths, and severe long-term disability [3]. *Streptococcus agalactiae* infections in tilapia result in high mortality rates and the annual economic loss in tilapia farming caused by this pathogen exceed 250 million USD [4]. Moreover, GBS caused a major invasive foodborne outbreak involving at least 146 people in Singapore [5]. The risk to multi-host cross infection and huge economic loss highlight the importance of developing vaccines to protect humans and animals from GBS [6].

Since the 1970s, GBS vaccines with GBS capsular polysaccharide alone have started to be investigated [7]. Maternal vaccination against GBS was demonstrated to be feasible in 1988, however the immunogenicity of plain polysaccharide vaccines was weak [8]. Then Dennis Kasper discovered that conjugate vaccines combining GBS polysaccharides with a carrier protein had the potential to elicit a more effective IgG response in comparison to polysaccharide alone [9]. In 2021, Judith Absalon and colleagues reported on a phase I/II clinical trial evaluating the safety, tolerability, and immunogenicity of a hexavalent glycoconjugate vaccine (serotypes Ia, Ib, II, III, IV, V), conjugated to a nontoxic mutant of diphtheria toxin (CRM197) [10]. Unfortunately, no GBS vaccine for human has been licensed up to now [11].

The principal difficulty in developing globally effective GBS vaccines is no cross protection among 10 serotypes [12]. So several conserved protective antigens of GBS, such as Sip [13], cell wall surface-anchored family proteins, CAMP factors, C5a peptidases, serine-rich repeat glycoproteins, etc, are considered as subunit vaccine candidates [14]. However, currently subunit vaccine with a single protective protein induced limited immune response and has not been a universally effective candidate against GBS.

Recently genomics, bioinformatics and proteomics technologies made possible to identify widely distributed conserved immunogenic proteins against pathogens. Furthermore, strategies are used to predict the antigenic epitopes, represent the minimal immunogenic region of a candidate protein and allow for predicting precisely directed immune responses [15,16]. The multi-epitope vaccines composed of different epitopes linked by ancillary linker have been demonstrated efficacy, specificity, safety and stability against various pathogens including *Leishmania protozoa* [17]*, human norovirus* [18]*, Staphylococcus aureus* [19] and *Shigella spp* [20]*.* However, studies on multi-epitope vaccines against GBS are limited. Hence, we sought to design a multiepitope vaccine against *S. agalactiae* (MVSA) based on antigenic candidate proteins screened by experimental data and immunoinformatic analysis. Moreover, when examining MVSA vaccine efficacy in mouse model, we observed MVSA, as well as anti-MVSA sera, could provide protection for mice in lethal GBS infection. Conclusively, our study showed MVSA provides a novel outcome to combat and control GBS infection and multiepitope vaccine was a promising strategy to prevent multi-serotype pathogenic bacterium infection.

## Materials and methodology

**Figure 1.**
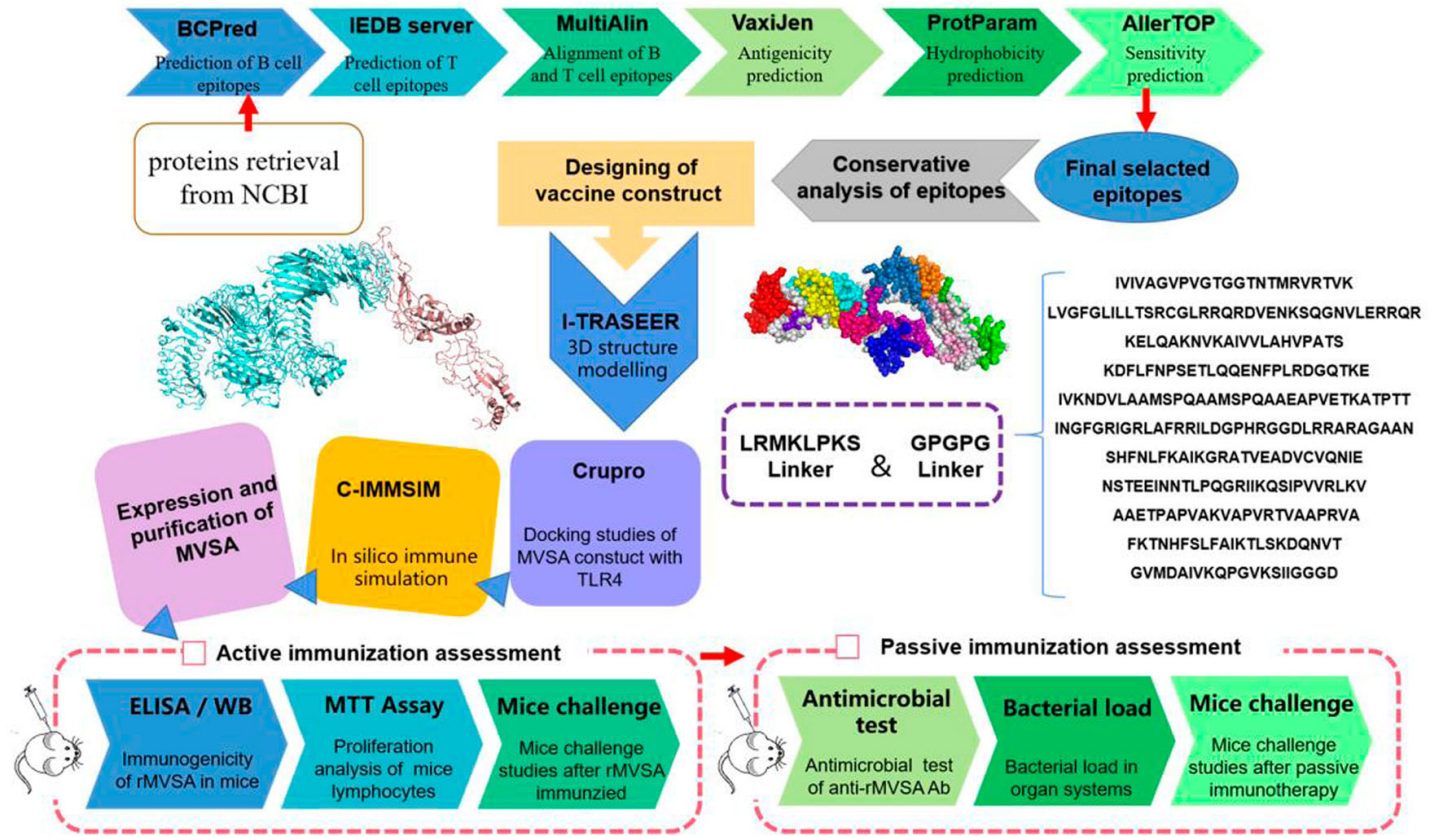
MVSA Vaccine Design. Immunoinformatic approaches implemented to design vaccine construct against GBS.

The comprehensive *in silico* analysis performed in this study to design a multiepitope vaccine against *S. agalactiae* (MVSA) is presented in Figure 1.

## Retrieval of protein sequences

In our previous studies, six proteins (NT5, OTC, BKD-E2, PK, GAPDH and PGK) from GBS had good immunogenicity through immunoproteomic method [21]. Meanwhile, candidate proteins that have been reported to be protective against GBS were summarized through bibliographic survey on the PubMed platform. The protein sequences retrieved from the genomic library file were used for further computational analysis to detect antigenic peptide sequences for vaccine design.

## Epitopes selection by affinity for B and T lymphocytes

B cell epitopes prediction was performed using two programs. ABCpred server at http://crdd.osdd.net was employed to predict liner B cell epitopes based on an artificial neural network [22]. In addition, the prediction of liner B cell epitopes was also performed by Bcpred (http://ailab.ist.psu.edu/bcpred/predict.html), and sequences with 20 amino acids as well as a 90% specificity threshold were considered [23]. Only proteins that both servers could predict out epitopes were chosen. The TEPITOOL(http://tools.iedb.org/tepitool/), which predicts peptides binding to MHC class I and class II molecules, was then used to examine these proteins for T cell affinity epitopes. The most prevalent MHC II HLAs molecules in humans, (DRB1 * 01: 01, DRB1 * 03: 01, DRB1 * 04:01, DRB1 * 07: 01, DRB1 * 11: 01, DRB1 * 13: 01 and DRB1 * 15: 01) were set to perform the server with at least 50% of allele binding [24]. B and T cell epitopes of candidate protein were aligned using MultiAlin (https://npsa-prabi.ibcp.fr), and peptides containing both B and T cell epitopes were selected. VaxiJen (http://www.ddg-pharmfac.net/vaxiJen/VaxiJen/VaxiJen.html) was used to predict the antigenicity of the peptides, and those with scores larger than 0.4 were thought to be possible antigens [25].

## Conservation among ten GBS serotypes

According to the 10 capsular genotypes of GBS, we downloaded the complete genomes of 138 GBS strains (containing serotypes Ia, Ib, II–VII) and 21 Scaffold genomes of GBS (including serotypes VIII and IX), and established a genomic database of 159 GBS strains covering 10 serotypes. Then, the distribution of epitopes in each GBS strain was counted and analysed by local tBLASTn. Meanwhile, based on the capsular gene cluster of 159 GBS strains, a phylogenetic tree was established by NJ method using MAGA X to analyse the conservation of epitopes in different serotypes.

## Design of the chimera protein

A chimeric protein was designed based on the previously selected peptides. First, the amino acid sequence of each epitope was submitted to the ProtParam tool program (https://web.expasy.org/protparam/) to predict the hydrophobicity index values [26]. According to the hydropathic index value, relatively hydrophobic epitopes were arranged in the middle and relatively hydrophilic epitopes were arranged at the ends. Furthermore, the ordered epitopes are connected by two “linkers.” The first spacer (GPPGPG) is inserted between the epitopes to keep them apart [27], and the second linker fragment (LRMKLPKS) is inserted at the N-terminal of each epitope to assist MHC II presentation [28]. Algpred (https://webs.iiitd.edu.in/raghava/algpred/submission.html) and VaxiJen were used to predict the allergenicity and antigenicity of the vaccine [29].

For the prediction of the tertiary protein structure of the MVSA, I-TASSER (https://zhanglab.ccmb.med.umich.edu/I-TASSER/) was used with the C-score value as the confidence score [30]. Furthermore, the PDB file of MVSA provided by I-TASSER was submitted to the GalaxyRefine server (https://galaxy.seoklab.org/) to refine the tertiary structure [31]. The ProSA-web (https://prosa.services.came.sbg.ac.at/prosa.php) and Ramachandran plot analysis (saves.mbi.ucla.edu/) were used to evaluated the refine the tertiary model [32,33].

## Molecular docking

The effective docking of the vaccine and receptors from immune cells contributes to product protective immune responses. Herein, the Cluspro 2.0 server (http://cluspro.bu.edu/login.php) was used for the docking analysis of MVSA with different immune cell receptors such as MHC I (PBD ID; 4u6y), MHC II (PDB ID; 5jlz), TLR 2 (PDB ID; 2z7x) and TLR3 (PDB ID; 3ulv) and TLR 4 (PDB ID; 4g8a) [34]. Then, the docking complexes were visualized using PyMOL software. To map the interacting residues between the vaccine and TLRs, PDBsum was used [35].

## Immune response simulation

To evaluate potential effectiveness of the vaccine, the website C-IMMSIM v10.1 (http://www.cbs.dtu.dk/services/C-ImmSim-10.1/) was used to predict possible immune responses following vaccine injection simulation [36]. We considered *in silico* administration of three injections were set at time steps of 1, 84 and 168, respectively (1-time step represents 8 h), and a minimum 30 days between two injections as described earlier [37]. The maximum value for simulation steps was set to 800 with the other stimulation parameters kept default.

## Cloning, expression and purification of rMVSA

The gene (1290 bp in length) encoding chimeric protein MVSA was synthesized and cloned into pET28a (+) vector. The vector was synthesized and cloned by Nanjing Kingsrui Company. The recombinant protein was then expressed in *E. coli* BL21[DE3] and affinity chromatography was performed using His Ni high performance column (GE Healthcare). The purified protein was analysed by SDS-PAGE on 12.5% gel which stained by Coomassie Blue. The protein concentration was quantified by BCA kit (Vazyme, China).

## Bacterial strains and culture

GD201008-001 was isolated from tilapia in our lab [38]. GBS strains ATCC 13813, ATCC 12403, ATCC BAA-611 obtained from ATCC. GBS human strains W58 and W78 were kindly provided by Chinese Center for Disease Control and Prevention. The GBS strains were firstly recovered on sheep blood agar (8%) at 37°C for 18 h under 5% CO_2_ atmosphere and then grow in THB. The *Escherichia coli* strain containing the recombinant chimeric gene was grown in Luria-Broth media with Kan^+^ at 37°C.

## Mice immunization and challenge

Mice were immunized by three subcutaneous injections of 20 μg/mouse recombinant MVSA prepared with Montanide ISA206 adjuvant (Seppic, France) at 10-day intervals. Mice injected with PBS in Montanide ISA206 were used as negative controls. The immunization procedure is shown in Figure 5(A). Two weeks after completion of the immunization procedure, mouse serum was collected by orbital blood sampling to assess antibody titre. GBS serotype V strain ATCC BAA-611 who had a moderated LD_50_ (1 × 10^7^ CFU/mouse, data not shown) was selected for further challenge experiments. Two weeks after the last immunization, the mice were injected intraperitoneally with virulent GBS strain ATCC BAA-611 20 × LD_50_ (2 × 10^8^ CFU/mouse). The mice were then monitored for 7 days for mortality and recorded for survival time.

## Antibody detection by enzyme-linked immunosorbent assay

After three immunizations, the indirect ELISA method was used to measure the anti-rMVSA antibody titres in the pooled serum. Endpoint titres were defined as the maximum dilution at which the mean absorbance OD450 was at least two times greater than the mean value of the negative control. Microliter plates were coated overnight at 4°C with 1.5 μg/mL of purified rMVSA in sodium carbonate buffer (pH 9.6) and each well was blocked with 200 μL of 5% skimmed milk in PBST for 1 h at 37°C. Then, serum samples were diluted in 96-well plates in 2-fold dilutions. Eight sample dilutions (from 1:100 to 1:204,800) were added and incubated at 37°C for 2 h. Subsequently, horseradish peroxidase-conjugated goat anti-mouse IgG antibody (1:5000) was added and incubated at 37 °C for 1 h. Add 200 μL PBST to each well for washing between each step and repeat 3 times. Antibody binding was detected by protein A-peroxidase conjugated (Sigma, P8651) followed by the substrate tetramethylbenzidine. Absorbance was measured at 450 and 570 nm according to the manufacturer's instructions.

## Western blot analysis

Western blot was performed to test the reactivity of r-MVSA hyper-immune serum with r-MVSA. Purified recombinant protein MVSA was separated on 12.5% SDS-PAGE gels and then electro-transferred onto PVDF membrane. After blocking the membrane in 5% skimmed milk, the membrane was incubated with a 1:1000 dilution of anti-r-MVSA serum. The membranes were then washed with PBS-Tween 20 (0.05%) followed by treating with a 1:2000 dilution of goat anti-mouse IgG conjugated to horseradish peroxidase (HRP)**.** Immunoreactive proteins were visualized by chemiluminescence using the Amersham ECL Plus Western blotting detection reagents (GE Healthcare).

## Lymphocyte proliferation assay and cytokine analysis

Seven days after the last immunization, three mice were taken from each group and the spleens were isolated by the following method [39]. The spleens were placed on sterile 200 mesh copper grids, washed with sterile PBS using a syringe, and splenocytes were collected. Then the resulting splenocytes were treated with ACK buffer (150 mM NH_4_Cl, 10 mM KHCO_3_ and 0.1 mM EDTA) to remove red blood cells. Splenocytes were resuspended in Dulbecco's modified Eagle medium (DMEM) supplemented with 10% heat-inactivated fetal bovine serum (Hyclone, Thermo scientific), 5 mM glutamine, 50 U/ml penicillin, 50 μg/ml streptomycin and 0.2% NaHCO_3_. The number of splenocytes used for stimulation studies was 10^6^ cells/well. Antigens (rMVSA) were used at concentrations of 10 µg/well. Experiments were performed in triplicate amounts. Proliferation was measured using MTT reagent (Sigma) after 72 h. The proliferation of lymphocytes was calculated as the stimulation index using the following formula. Stimulation index = OD_570_ with antigen/OD_570_ without antigen. Lymphocytes from different wells were collected simultaneously and RNA was extracted for RT-qPCR to estimate cytokines (IL-2, IL-4, IL-10 and TNF-α).

## *In vitro* antimicrobial activity testing

To determine the antibacterial activity of anti-rMVSA antibodies against GBS, an in vitro antimicrobial assay was performed. Briefly, single colonies of different serotypes GBS strains were picked in THB and cultured overnight. The strains were transferred to 5 mL THB according to 1:100, and incubated at 37°C with shaking at 180 rpm to log phase (OD_600 _= 0.6∼0.8). Add 100 μL of dilutions of different serotypes of GBS diluted 50-fold with THB solution to the microplate, and count bacterial numbers on THB agar (THA). The hyperimmune serum of MVSA and negative serum were diluted 50 times with THB, which was added 100 μL to each microplate with different bacteria dilutions. After incubation at 37°C for 2 h, the different wells were serially diluted and counted bacteria on THA.

## Passive immunotherapy

In 4-week-old female ICR mice (*n* = 10), the prophylactic and neutralizing effects of high immunity serum *in vivo* were determined by intraperitoneally injecting 200 μL of anti-rMVSA serum and 24 h later challenging with 20 × LD_50_ (2 × 10^8^ CFU/mouse) of GBS ATCC BAA-611. The control group (*n* = 10) was injected with the same amount of PBS alone and then challenged with the same amount of lethal dose. The mice were observed for 7 days and mortality was recorded.

## Bacterial load in organ systems

Mice received passive immunotherapy 24 h in advance and then challenged with a lethal dose of GBS ATCC BAA-611 (2 × 10^8^ CFU/mouse). After a 9-hour observation, mice were sacrificed by CO_2_ sedation followed by cervical dislocation, and the various organs were separated and collected. Blood was collected from the eyes of the mice and counted bacteria on THA after dilution with PBS. The spleen, liver and brain were further excised and transferred into sterile pre-weighed MP tubes. Different tissues were macerated with a tissue grinder, and a portion of the resultant homogenate was diluted in THA to count bacteria, while the remainder served as a sample for RNA extraction.

## Real-time qPCR analysis

RNA was extracted for reverse transcription quantitative real-time quantitative PCR (RT-qPCR) using TRIzol (Vazyme, China), as directed by the manufacturer. HiScript II Q RT SuperMix (Vazyme, Nanjing, China) was used to create cDNA from 1 g total RNA. RT-qPCR analysis was performed using an using ChamQ Universal SYBR qPCR master mix (Vazyme, China) and the QuantStudio 6 Flex real-time PCR system. The gene GAPDH was used as an internal control and was run concurrently to standardize the input cDNA. The primer sequences used in the study were provided in supplementary Table S1.

## Results

### Selection and characterization of proteins

Our selected 15 proteins were experimentally proven protection against GBS and summarized in Table 1, among which six candidates (NT5, OTC, BKD-E2, PK, GAPDH and PGK) were identified as immunoreactive proteins in our previous immunoproteomics study [21].

**Table 1.** The 15 candidate proteins against GBS.

Number	Protein	Protein note	Protein acession
1	PK	pyruvate kinase	WP_001042781.1
2	FbsA	fibrinogen-binding surface protein A	WP_000482176.1
3	NT5	5'-nucleotidase family protein	WP_000726930.1
4	AP1-2b	PI-2b ancillary protein 1	WP_000913272.1
5	BKD-E2	branched-chain alpha-keto acid dehydrogenase subunit E2	WP_000257565.1
6	GAPDH	glyceraldehyde 3-phosphate dehydrogenase	WP_000260656.1
7	Srr1	serine-rich repeat protein	WP_000039461.1
8	BibA	Immunogenic bacterial adhesin	WP_001063288.1
9	Sip	surface immunogenic protein	WP_000783424.1
10	ACP	Alpha C protein	WP_000489957.1
11	BCP	Beta C protein	WP_000477136.1
12	BP-2b	PI-2b backbone protein	WP_000616199.1
13	PGK	phosphoglycerate kinase	WP_001096753.1
14	Lrrg	leucine-rich repeat domain-containing protein	WP_000162162.1
15	OTC	ornithine carbamoyltransferase	WP_000195399.1

## Prediction of epitopes with B and T lymphocytes

**Figure 2.**
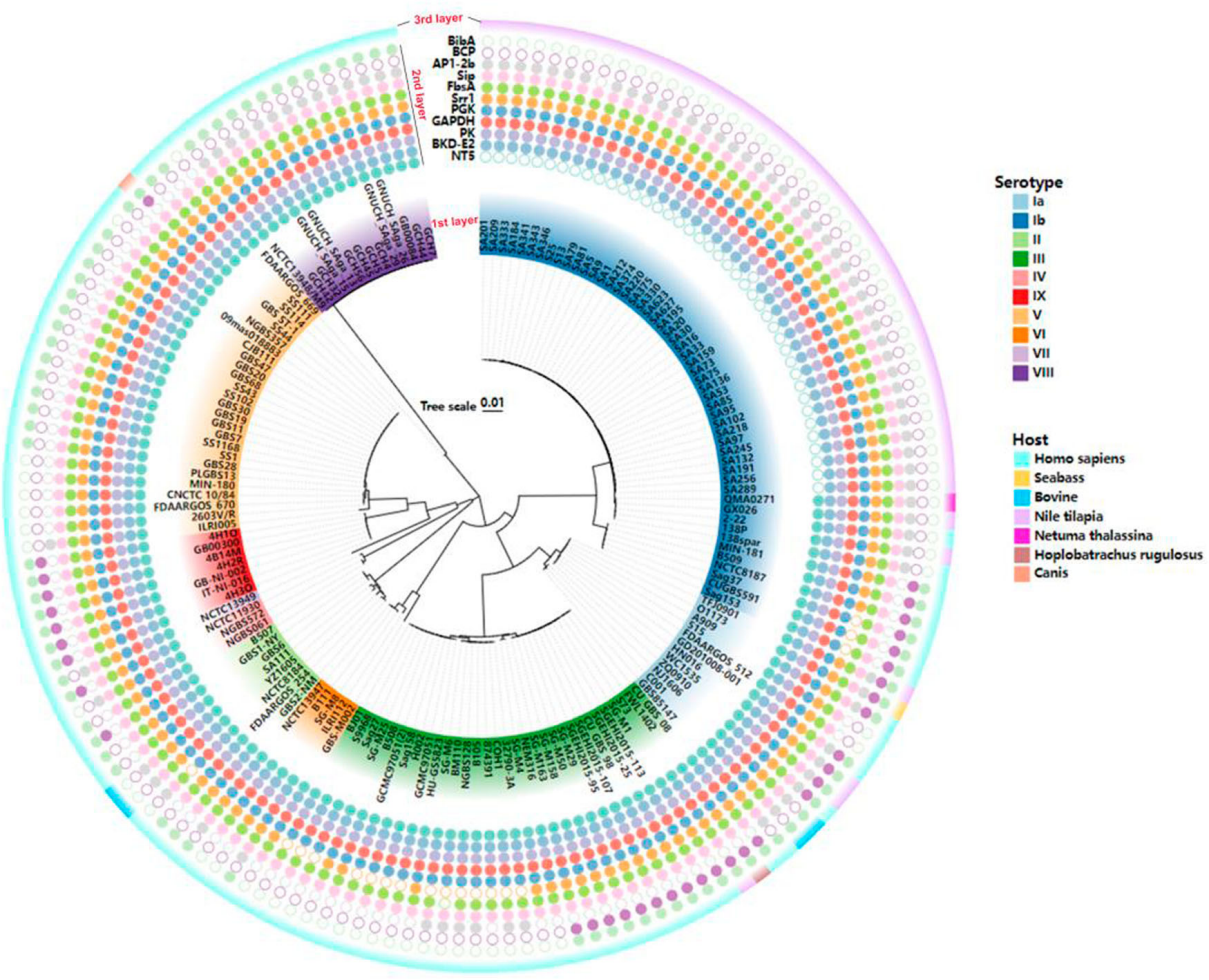
Conservative analysis of predicted 11 epitopes on 10 serotypes of GBS. The phylogenetic tree was reconstructed based on the CPS cluster from 159 GBS genomes. The first layer showed strain ID and the serotypes. The second layer showed the distribution of 11 epitopes while the filled circles represented the epitope existed in the corresponding GBS strain, and hollow circles meant the epitope was not distributed in the corresponding GBS strain. The third layer showed the host source of GBS strains. The first layer to the third layer is from inward to outward.

The candidate proteins were predicted B-cell epitopes by BCPred, ABCPred and T-cell epitopes by IEDB TEPITOP, respectively, and only the epitopes which recognized by both T cells and B cells were selected. However, among 15 proteins, no valid B-cell epitope prediction was obtained for OTC. The epitopes with predicted antigenicity greater than 0.4 were selected, and then two long-chain peptide epitopes with lower antigenicity were removed, such as ACP and BP-2b, and the final selected epitopes were the 11 epitopes shown in Table 2. To verify the ubiquity of the 11 predicted antigenic peptides among GBS, they were submitted respectively to local BLAST based on a database containing 159 GBS genome (containing 138 completed and 21 scaffold genomes) covering 10 serotypes*.* As shown in Figure 2, three epitopes (PK, GAPDH and Sip) were consistently present in all GBS strains. Furthermore, at least 7/11 epitopes coexisted in each GBS strain, and even all 11 epitopes were present in serotype Ia GBS strains. These results indicated that these 11 epitopes could cover 10 GBS serotypes *in silico*, suggesting the potential to protect the host from the invasion of multi-serotype GBS.

**Table 2.** Selected epitopes with affinity for B and T lymphocytes and antigenic score.

Number	Protein	Epitope	Antigenic score
1	PK	IVIVAGVPVGTGGTNTMRVRTVK	1.1044
2	FbsA	LVGFGLILLTSRCGLRRQRDVENKSQGNVLERRQR	0.7712
3	NT5	KELQAKNVKAIVVLAHVPATS	0.7422
4	AP1-2b	KDFLFNPSETLQQENFPLRDGQTKE	0.6902
5	BKD-E2	IVKNDVLAAMSPQAAMSPQAAEAPVETKATPTT	0.6568
6	GAPDH	INGFGRIGRLAFRRILDGPHRGGDLRRARAGAAN	0.6114
7	Srr1	SHFNLFKAIKGRATVEADVCVQNIE	0.603
8	BibA	NSTEEINNTLPQGRIIKQSIPVVRLKV	0.5651
9	Sip	AAETPAPVAKVAPVRTVAAPRVA	0.5648
10	BCP	FKTNHFSLFAIKTLSKDQNVT	0.4748
11	PGK	GVMDAIVKQPGVKSIIGGGD	0.4427

## Design of multi-epitope protein and molecular docking

**Figure 3.**
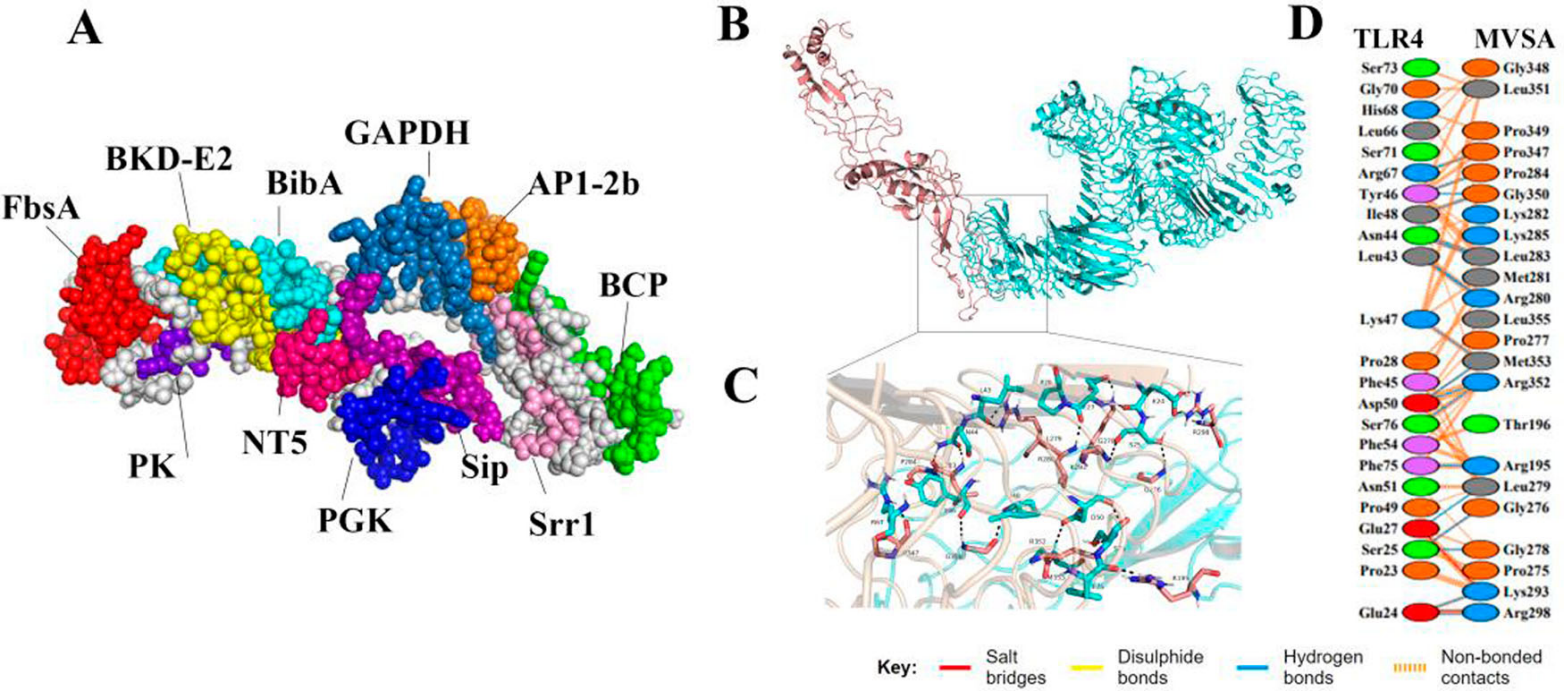
MVSA 3D structural analysis and molecular docking (A) The tertiary structure of the MVSA. The various epitopes were indicated by different colours; the white region represented the linkers “GPGPG”and “LRMKLPKS.” (B) Diagram of docking mode of the MVSA-TLR4 complex. (C) Docked conformation and hydrogen bond interaction map of MVSA (shown in blue) to TLR4 (shown in pink), and the black dotted lines referred to hydrogen bonds. (D) The interacting residues between docked MVSA and TLR4.

**Figure 4.**
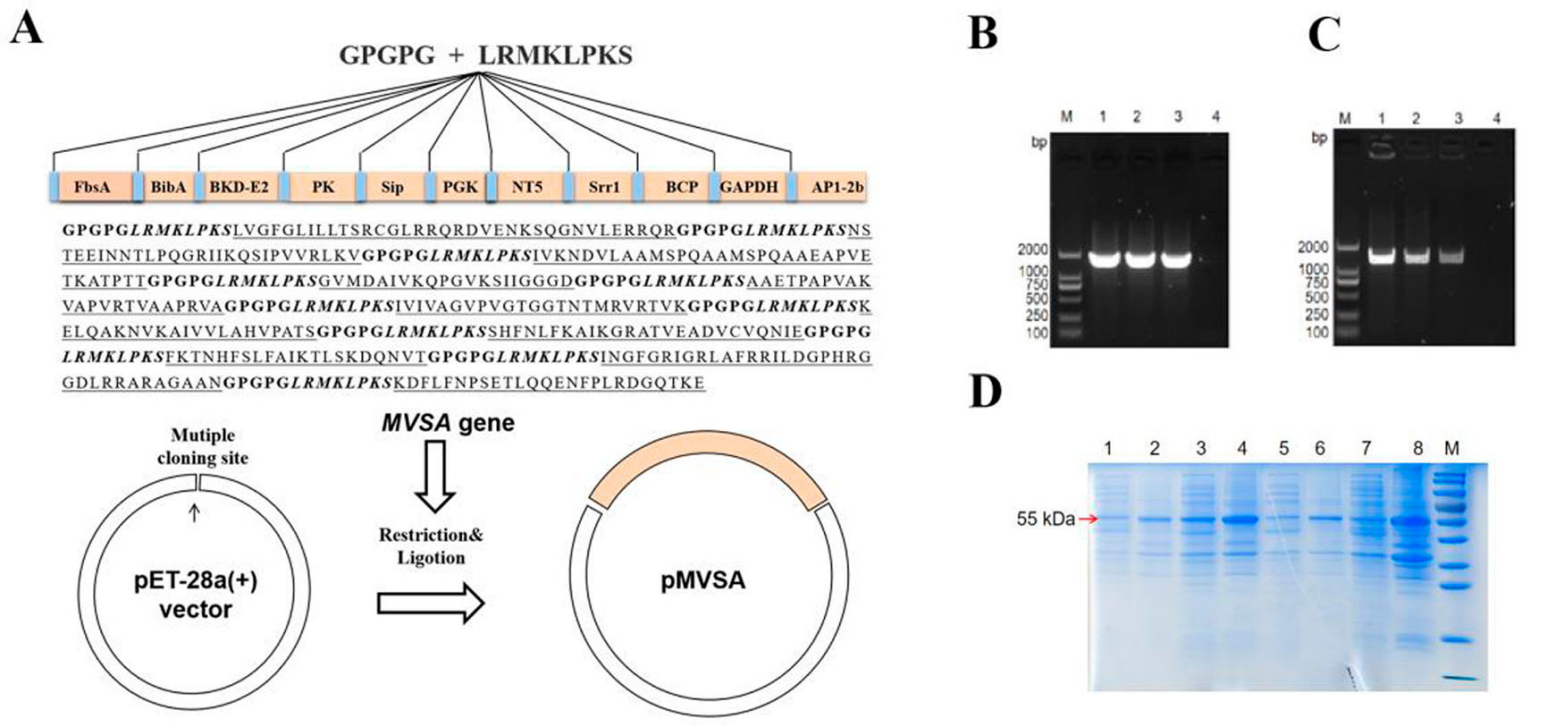
*In silico* cloning of the MVSA in the pET28a (+) and expression. (A) Cloning and expression of MVSA in pET28a(+) vector. The 11 epitopes were fused together in proper order by the appropriate linkers. Agarose gel showing MVSA was amplified by primers MVSA-P1/P2 (B) and T7/T7RVERS(C). Lanes 1–2: pET-28a-MVSA-BL21; Lane 3: pET-28a-MVSA; Lanes 4: negative control. (D) SDS-PAGE gel showing rMVSA expression after sonication. rMVSA was induced by 1 mM IPTG for incubation 5 h at 37°C (lanes 1–2), 0.5 mM IPTG for incubation 14 h at 28°C (lanes 3–4), 1 mM IPTG for incubation 16 h at 16°C (lanes 5–6) and 0.5 mM IPTG for incubation 16 h at 37°C (lanes 7–8). rMVSA in Lanes 1, 3, 5, 7 were expressed in the supernatant, and rMVSA from lanes 2, 4, 6, and 8 were in inclusion bodies.

According to the hydrophilicity gradually increasing from the middle to the flanks, the 11 epitopes were connected by “GPGPG” and “LRMKLPKS” to link the 11 epitopes to obtain the final protein sequence of MVSA (Figure 4(A)). The antigenicity of MVSA was predicted to be 1.1909, and it was not allergenic*.* Five 3D models of MVSA were constructed by I-TASSER, and the model with −1.45 C-score is the best for further refinement. The refined 3D structure of MVSA through GalaxyWeb server was shown in Figure 3(A) with increased in the Ramachandran plot's scores: 76.5% of residues in most favoured regions, 20.4% in additional allowed regions, 0.9% of residues in generously allowed regions, and 2.2% in disallowed regions (Figure S1). Also, the Z-score of the refined model was estimated −4.16 (Figure S2). Docking process between vaccine and TLR4 was evaluated by Cluspro 2 program. As shown in Figure 3(B), the best-docked model was selected according to the biochemical criteria of protein ligand and TLR4. The MVSA–TLR4 binding interface was shown in Figure 3(C,D): 13 hydrophilic amino acids (G278, R195, G276, L279, R280, L283, P284, K293, R298, P347, G350, R352, M353) of TLR4 formed intensive hydrogen-bond networks with 12 amino acids (E24, S25, E27, P28, L43, N44, L46, I48, D50, R67, F75, S76) of MVSA (Figure 3(C)), and a total of 23 MVSA residues coupled with 23 residues of chain A from TLR4 molecule (Figure 3(D)). Additionally, molecular docking modes between the vaccine and TLR2(Figure S3), TLR3(Figure S4), MHC I (Figure S5) and MHC II (Figure S6) were also performed in this study, and the binding interface diagrams of the complexes among them are shown in the supplementary material, demonstrating that the MVSA had a good affinity for TLRs and MHC molecules.

## *In silico* immune simulation

*In silico* immune simulation was performed to characterize the immune profile of the designed multi-epitopes vaccine. The simulated immune response of MVSA was extensively activated, and there was a potential simulated increase in antibody titre after injection (Figure S7A). The populations of B cell, T cell and NK cell were also increased considerably (Figures S7BCDE). The simulated cytokine responses were also predicted, showing reliable and vigorous response following injection (Figure S7F). These results indicated that MVSA could effectively elicit a strong immune response *in silico*.

## Expression and purification of recombinant chimeric protein MVSA

MVSA containing a total of 430 amino acids was artificially synthesized and linked to pET-28a (+) to construct the recombinant plasmid pET-28a-MVSA (Figure 4(A)). Sequencing of the pET-28a-MVSA revealed no deletion or point mutation compared to the expected sequence. Then pET-28a-MVSA was successfully transformed into *E.coli* BL21 (DE) (Figure 4(B,C)). The SDS-PAGE electrophoresis (Figure 4(D)) showed the recombinant protein MVSA (rMVSA) was 55 kDa in size in both supernatant and precipitate after sonication while the expression of soluble rMVSA was the highest in the supernatant induced by 0.5 mM IPTG for incubation 14 h at 28°C.

## Immunogenicity of rMVSA in mice

**Figure 5.**
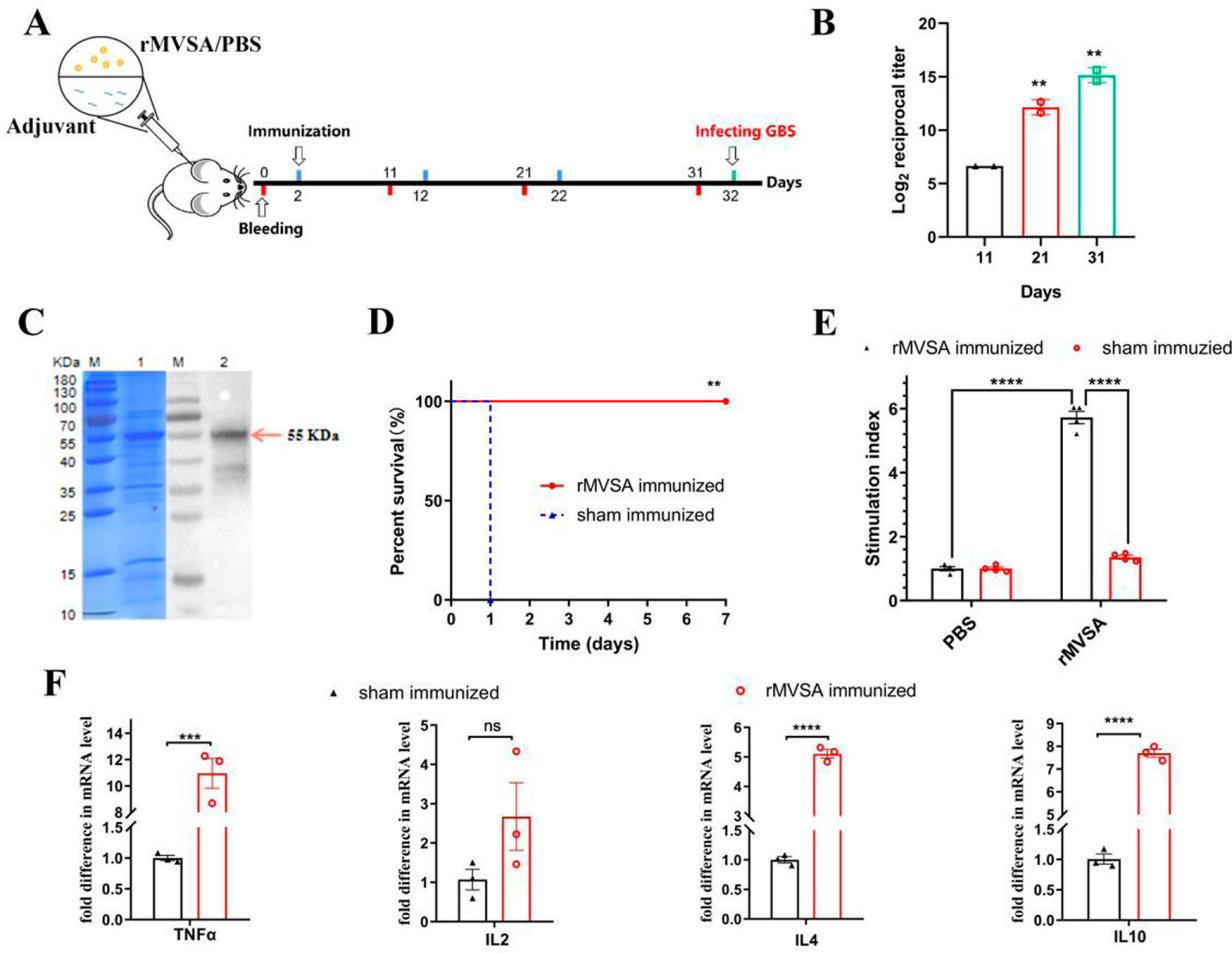
MVSA active immune protection assessment. Timeline for active immunization of MVSA vaccine regimen, comprising of an initial prime, 2 boosts, 4 bleeding and final infecting. Mice were stratified into rMVSA immunized group or sham immunized control group. (B) The antibody titres induced by rMVSA at each immunization. (C) rMVSA protein was purified with Ni-NTA agarose shown in the left SDS-PAGE gel and the reactivity of anti-rMVSA serum with rMVSA was tested in western blot on the right. (D) The survival curves for rMVSA-immunized and sham-immunized mice challenged with 20×LD_50_ of ATCC BAA-611 strain (**, *p* <0.01). (E) Proliferation of lymphocytes isolated from rMVSA-immunized and sham-immunized mice and treated with rMVSA6 protein for 72 h. (F) Cytokine levels of splenocytes isolated from immunized mice after stimulation with rMVSA. Significance (P)-value summary analysed by Unpaired two-tailed Student’s *t*-test (*, *p* < 0.05; **, *p* < 0.01; ***, *p* < 0.001; ****, *p* < 0.0001; ns, no significance).

The immunization procedure for mice was shown in Figure 5A. Anti-rMVSA polyclonal antibodies in mice were shown to a gradual increase with boosted immunization by an indirect ELISA (Figure 5(B)). After three immunizations, the endpoint antibody titre reached 1:25,600, whereas negative sera did not react with rMVSA in ELISA. Meanwhile, a specific band at around 55 kDa on PVDF membrane (Figure 5(C)) was similar to that in SDS-PAGE electrophoresis, indicating that anti-rMVSA polyclonal antibodies had specific recognition with rMVSA protein.

## Proliferation and cytokine analysis of rMVSA-immunized mouse lymphocytes

The rMVSA antigen induced a significant proliferation of splenocytes (whole lymphocytes) from rMVSA immunized mice while no significant proliferation was observed in splenocytes from sham-immunized mice (Figure 5(E)). In addition, we assessed the levels of cytokines in the conditioned medium after lymphocyte proliferation and found that rMVSA-sensitized lymphocytes produced significantly more cytokines TNFα, IL-4, and IL-10 compared to lymphocytes from sham immunized mice (Figure 5(F)).

## Mice challenge studies

After three immunizations on the mouse model, we evaluated the immunoprotective effect of rMVSA against GBS infection. When I.P injected with 20 × LD_50_ of strain ATCC BAA-611 (2 × 10^8^ CFU/mouse), mice in the rMVSA immunized group were no symptom and 100% survival during 7 days observation, while all of the sham immunized mice died in 1 day. The result showed that the rMVSA provided 100% protection for mice against lethal GBS infection (Figure 5(D)).

## Antibacterial activity of rMVSA polyclonal antibodies

**Figure 6.**
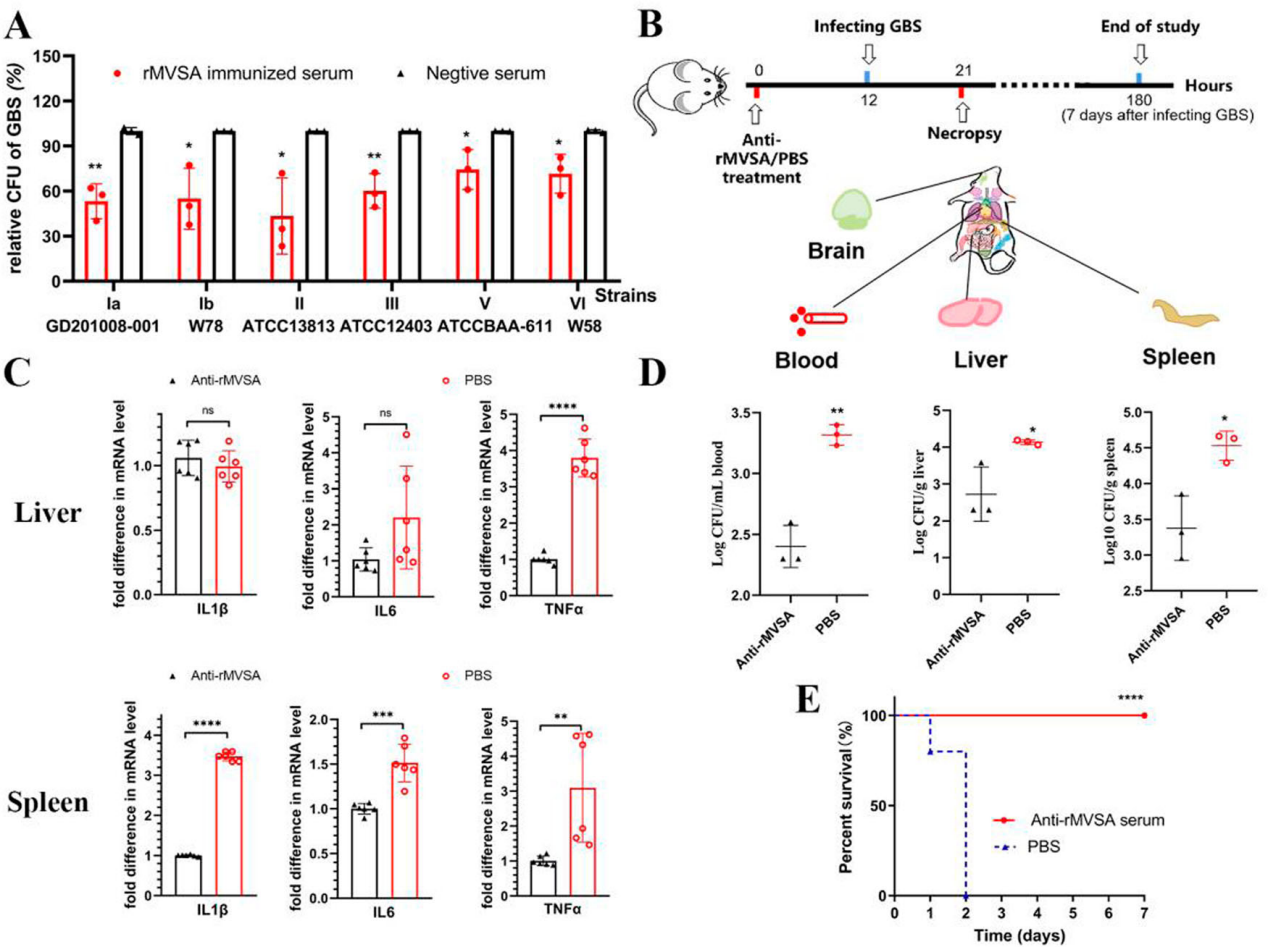
MVSA passive immune protection assessment. (A) The antibacterial activity of anti-rMVSA serum against various serotypes GBS strains. (B) Schematic representation of passive immune protection schedules of anti-MVSA serum, containing immunization, infecting and necropsy. Mice were stratified into anti-rMVSA serum group or PBS control group. (C) Cytokine transcription Levels in two organs (Liver and Spleen) of mice from different groups. (D) Viable bacterial counts for tissues from different groups of challenged mice. (E) The survival curve for anti-rMVSA serum and PBS groups mice challenged with 20 × LD_50_ of ATCC BAA-611 strain (****, *p* <0.0001). Significance (P)-value summary analysed by Unpaired two-tailed Student’s t-test (*, *p* < 0.05; **, *p* < 0.01; ***, *p* < 0.001; ****, *p* < .0001; ns, no significance).

To assess the antibacterial activity of rMVSA antibodies, the antibody inhibition assay was utilized *in vitro*. The rMVSA polyclonal sera were mixed with six crucial serotypes GBS strains (Ia, Ib, II, III, V, VI) and the bacteria replication were examined after incubation 2 h by viable colony counts**.** The results showed rMVSA immunized serum significantly inhibited the growth of different serotype GBS strains (Figure 6(A)).

## Anti-rMVSA antibody provides protection for mice

To evaluate the protection of anti-rMVSA antibody against GBS infection *in vivo*, three experiments were utilized after anti-rMVSA treatment, including RT-qPCR for organ cytokines, GBS distribution in mice organ, and passive immune challenge assay in mice (Figure 6(B)). RT-qPCR for organ cytokines showed that the transcriptional level of IL-1β, IL-6 and TNFα in the spleen from anti-rMVSA treatment group were significantly higher than those from PBS treatment group. The transcriptional level of TNFα in the liver from anti-rMVSA treatment group was significantly higher, but IL-1β and IL-6 were exception (Figure 6(C)). Meanwhile, mice in the anti-rMVSA treatment group had less bacteria amount in blood, liver and spleen than in the PBS treatment group after the 20 × LD_50_ ATCC BAA-611 challenge while no GBS distributed in the brain of mice from both groups Figure 6(D). Importantly, the passive immune challenge in mice showed that 100% of the challenged mice survived beyond the observation period in anti-rMVSA treatment group while all of the mice in the untreated group died within 2 days after challenge (Figure 6(E)). These results confirmed that the rMVSA immune serum could inhibit GBS colonization *in vivo*.

## Discussion

Up to date, GBS still plays a main role in cow mastitis, tilapia meningoencephalitis, neonatal meningitis, and puerperal sepsis in pregnant women, while poses a serious burden to the farming industry and public health. With the increase in GBS resistance to existing antibiotics [40], vaccine design and production has become the most effective measure to prevent GBS. Compared to polysaccharide vaccines, protein-based vaccine formulations are lower cost to produce and have broader coverage. Therefore, previous studies have attempted to identify surface proteins expressed in all GBS serotypes as vaccine antigen candidates. On this basis, numerous protective antigens, expressed chimerically or combined with other immune stimulants, have been studied as alternative vaccine candidates. In this study, 11 highly conserved proteins in GBS based on experimental data were selected to design a multi-epitope vaccine MVSA to against all GBS serotypes via immunoinformatic analysis, and MVSA successfully provided the 100% protection for mice in both active and passive immune protection assays.

Recent advances in computational biology and immunoinformatics can greatly facilitate the designing safe and efficient vaccines in a time and cost-effective manner [41]. For instance, a vaccine approved for commercialization [Bexsero®] via reverse vaccinology has been used in humans to prevent *Neisseria meningitidis* serogroup B infection, demonstrating the value of this approach [42]. Moreover, a multi-epitope subunit vaccine designed by immunoinformatics software was shown to be a cost-effective candidate against *Acinetobacter baumannii* [43]. Here, we used immunoinformatic methods combined with experimental data to design MVSA for vaccine effectiveness.

To make the multi-epitope vaccine more effective, the epitopes selection and connection are critical. Firstly, both B cell epitopes responsible for humoral immune response and T cell epitopes inducing cellular immunity should be considered when preparing epitope-based vaccines. The selection of a single epitope may cause immune failure due to insufficient stimulation of immune response. Therefore, we selected miscellaneous peptide containing both B and T cell epitopes from each candidate protein. Moreover, the proper use of linkers could form a sturdy frame and improve expression and biological activity of the multi-epitope recombinant protein. GPGPG makes distance between the epitopes to prevent the creation of neoepitopes and changes in the final construct, facilitating the processing and presentation of each correct epitope as well as the ability to induce humoral immune response [27]. Moreover, an Ii-Key fragment (LRMKLPKS) from the murine invariant chain protein has been reported to enhance MHC II presentation [28]. A multi-epitope vaccine against *Streptococcus pneumoniae* was utilized GPGPG and LRMKLPKS and induced a high immune response [24]. Using GPGPG and LRMKLPKS to separate five T cell epitopes isolated from *Coccidioides immitis* and *C. posadasi* to construct a recombinant epitope protein which showed a significant reduction of fungal burden and prolongation of survival compared to no vaccinated mice [44]. Consistently, the two linkers used to connect epitopes help MVSA to effectively expose each epitope as shown in the 3D structure, which is beneficial to the recognition of immune cells and effective antibody against GBS invasion.

The most essential assessment of multi-epitope vaccine is the corresponding immune response could availably reduce illness or even death when pathogens re-invade the host. A multi-epitope vaccine containing 5 antigenic peptides of GBS activate antibody production and demonstrated promising protection against bacterial disease in tilapia [45]. Hereon, we not only successfully predicted the potential efficacy of MVSA *in silico* against 10 serotypes GBS, but also successfully evaluated the protection provided by MVSA on the mouse model. Furthermore, considering that the LD_50_ of different serotypes of GBS on mice is quite different, and even some serotypes cannot choose a suitable challenge dose to perform the immune protection test [46], we used anti-MVSA antibody to evaluate the growth inhibitory effect on six prevalent serotypes (Ia, Ib, II, III, V, VI).

To sum up, these findings indicated that MVSA was an ideal anti-GBS vaccine candidate. Furthermore, our rational epitope-prediction workflow could be applicable for multivalent vaccine development for other pathogenic diseases as well.

## Supplementary Material

Supplemental Material
