# Supplementary material for: Development and evaluation of a multi-epitope subunit vaccine against group B Streptococcus infection
Source: Emerg Microbes Infect. 2022 Sep 29;11(1):2371–82. doi: 10.1080/22221751.2022.2122585 (PMC9543083; doi:10.1080/22221751.2022.2122585)
Supplement: Supplemental Material [file TEMI_A_2122585_SM6100.docx]

| **Primers** | **Sequence（5’-3’）** | **Annotate** |
| --- | --- | --- |
| MVSA-P1 | GGGCCAGGTCCCGGATTAAGGA | primers for pET-28a-MVSA |
| MVSA-P2 | TTCTTTGGTCTGACCATCTCT |  |
| T7 | TAATACGACTCACTATAGGG |  |
| T7 Ter | TGCTAGTTATTGCTCAGCGG |  |
| GAPDH-F | TGCACCACCAACTGCTTAG | primers for qRT-PCR |
| GAPDH-R | GGATGCAGGGATGATGTTC |  |
| IL-1β-F | TCCAGGATGAGGACATGAGCAC |  |
| IL-1β-R | GAACGTCACACACCAGCAGGTTA |  |
| IL-6-F | CCACTTCACAAGTCGGAGGCTTA |  |
| IL-6-R | GCAAGTGCATCATCGTTGTTCATAC |  |
| TNFα-F | GGTGCCTATGTCTCAGCCTCTT |  |
| TNFα-R | GCCATAGAACTGATGAGAGGGAG |  |
| IL-2-F | GCGGCATGTTCTGGATTTGACTC |  |
| IL-2-R | CCACCACAGTTGCTGACTCATC |  |
| IL-4-F | ATCATCGGCATTTTGAACGAGGTC |  |
| IL-4-R | ACCTTGGAAGCCCTACAGACGA |  |
| IL-10-F | CGGGAAGACAATAACTGCACCC |  |
| IL-10-R | CGGTTAGCAGTATGTTGTCCAGC |  |

**Table S1 The primers in the study**


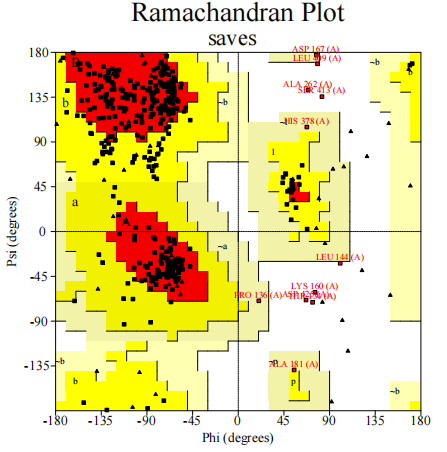


**Figure S1 Ramachandran plots of selected models of MVSA.** The different colors in the graphs represent the followings, black square (torsion angles of the polypeptide), red color (secondary structure elements), yellow color (favored region), pale yellow color (allowed region), and white color (disallowed region).


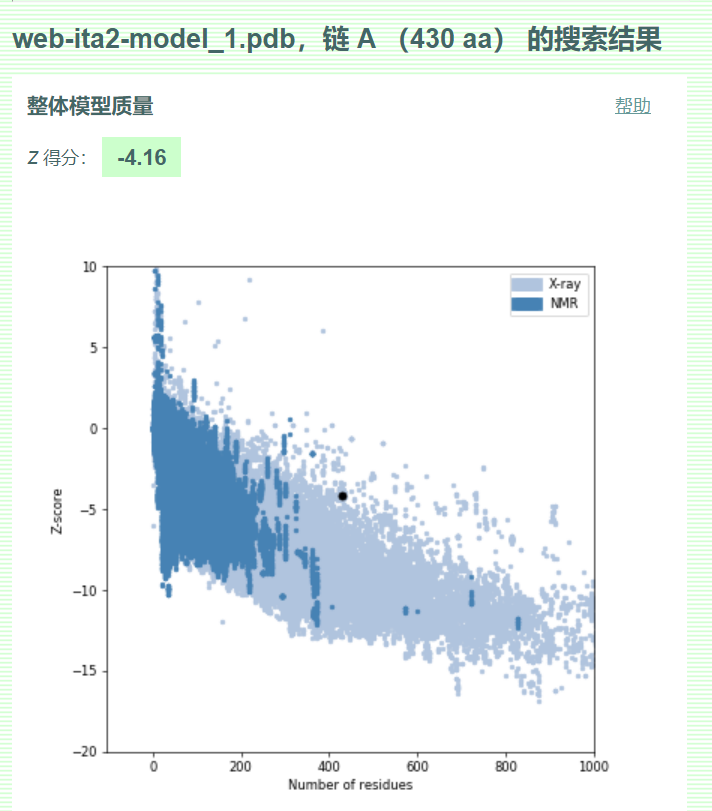


**Figure S2 ProSA-web evaluation of the vaccine structure.** ProSA-web results indicated a Z-score was ‒4.16.


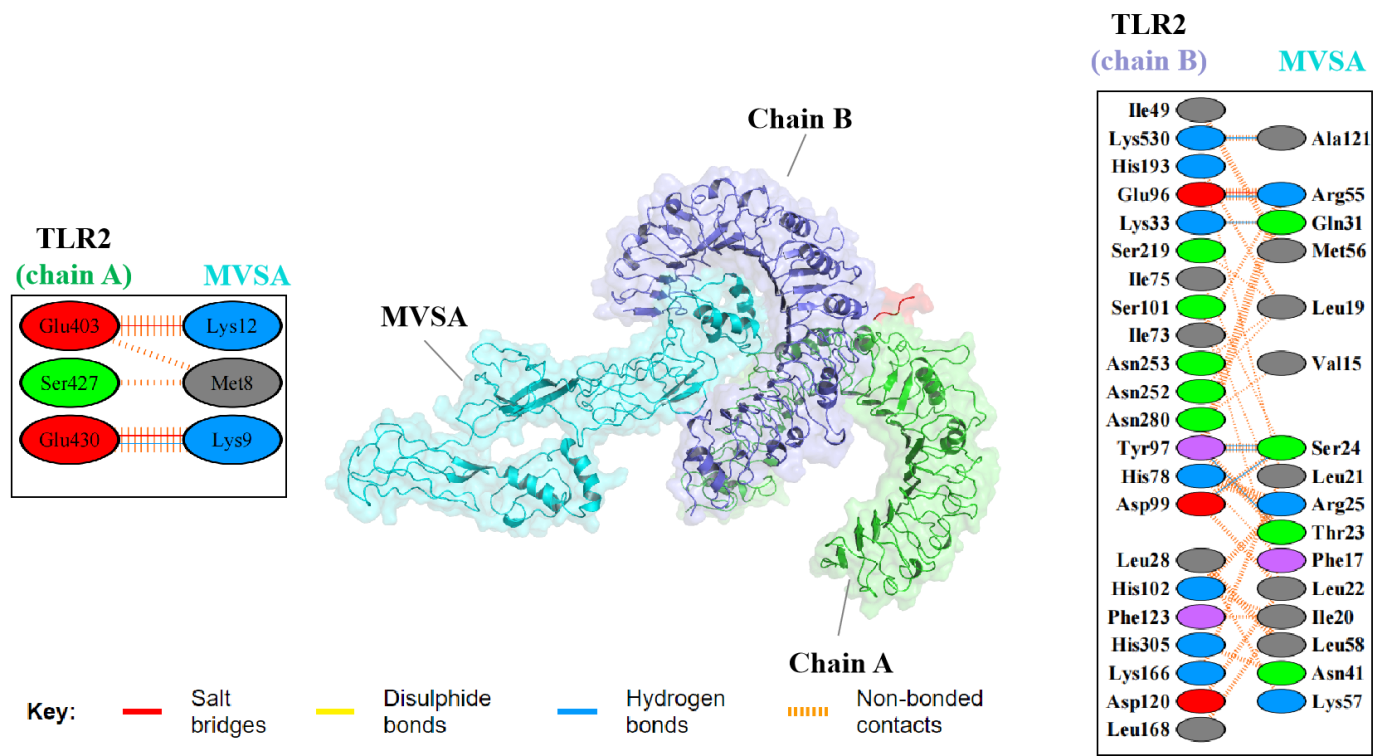


**Figure S3 Intermolecular binding mode and residue-level chemical interactions of MVSA-TLR2.**


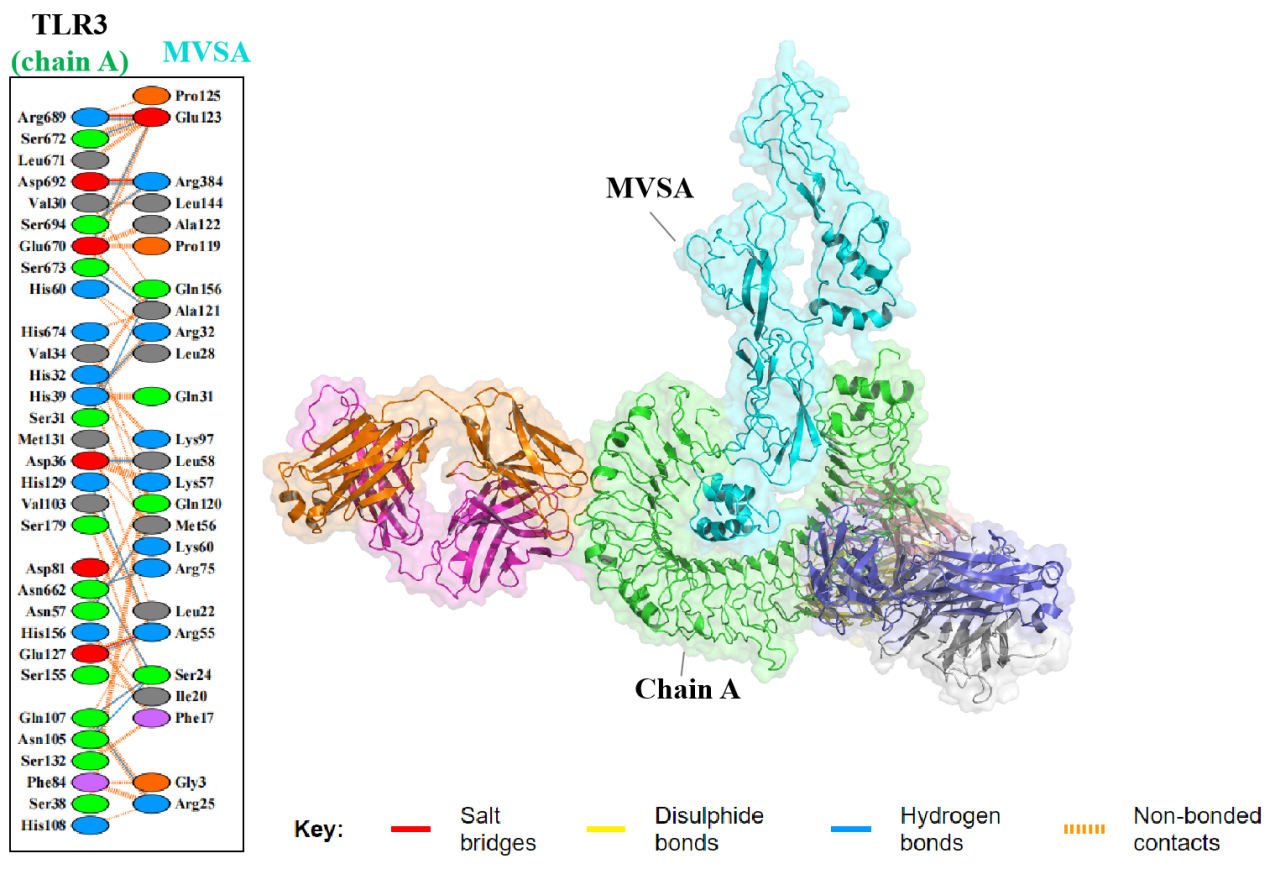


**Figure S4 Intermolecular binding mode and residue-level chemical interactions of MVSA-TLR3.**


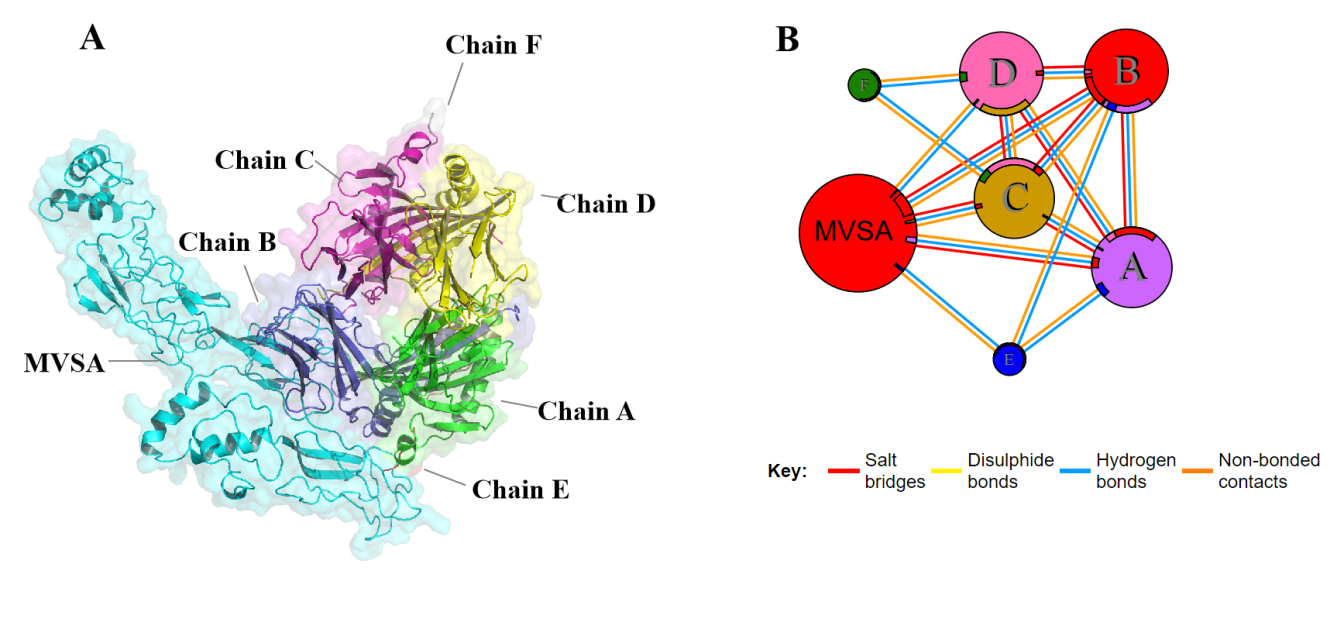


**Figure S5 MVSA-MHC II binding conformation and interaction analysis.** (A)Intermolecular binding mode and residue-level chemical interactions of MVSA-MHC II. (B) Schematic diagram of interactions between MVSA and MHC II protein chains. The extent of the interface region on each chain is represented by a colored wedge whose color corresponds to the color of the other chain and whose size signifies the interface surface area.


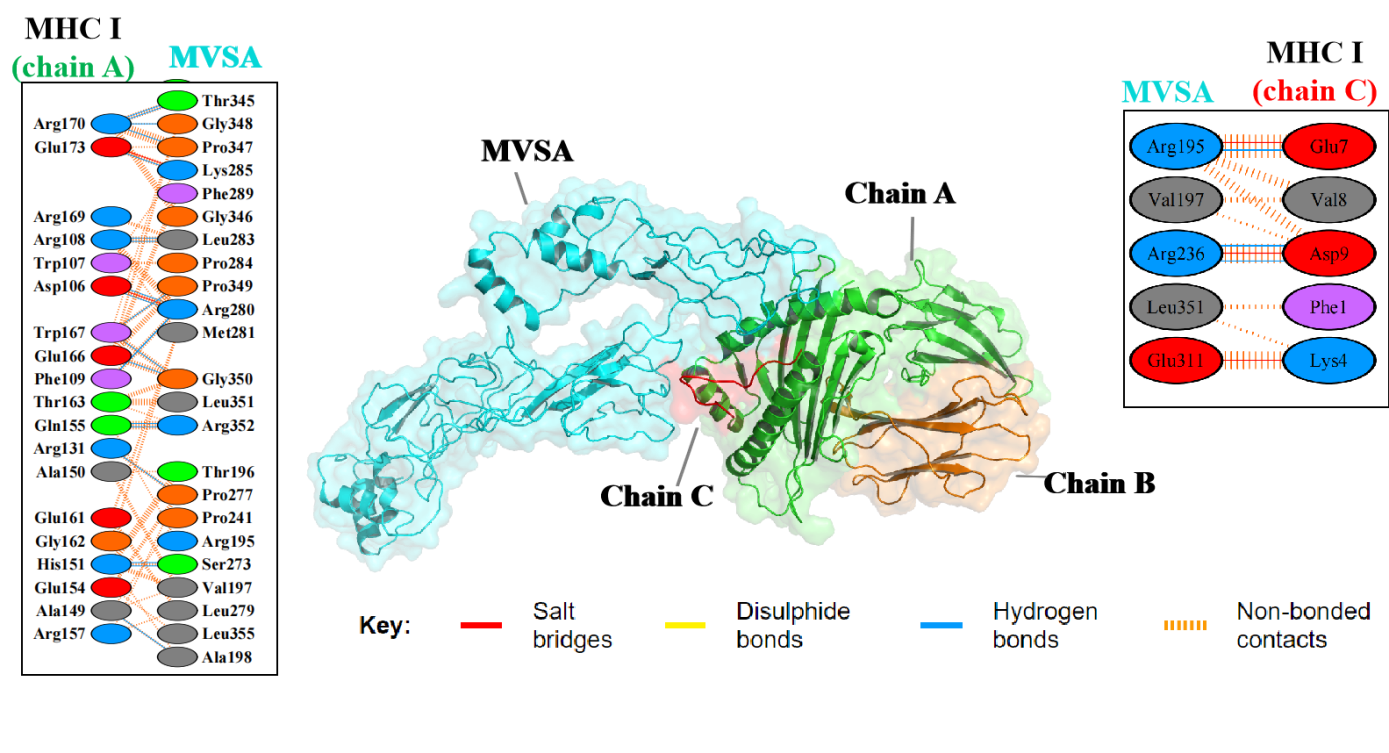


**Figure S6 Intermolecular binding mode and residue-level chemical interactions of MVSA-MHC I.**

**
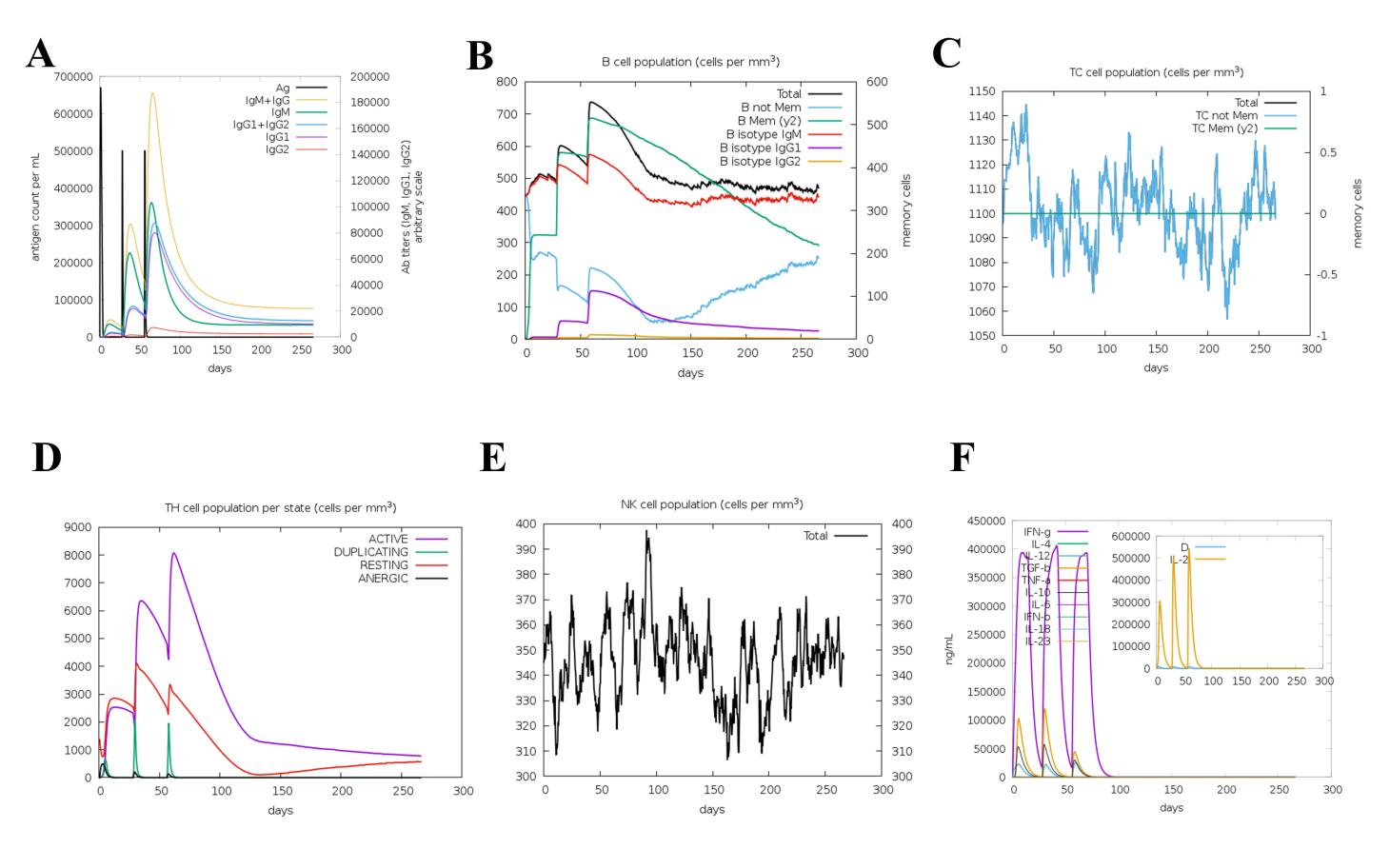
**

**Figure S7 The *in silico* immune simulation results.** (A) Immunoglobulin levels with respect to antigen concentration. (B) B-cell population. (D) TC (cytotoxic) cell population. (E) TH (helper) cell population. (E) The population of NK cells (F) Cytokine level in response to the vaccine.
